# HIV and Antiretroviral Therapy Are Independently Associated with Cardiometabolic Variables and Cardiac Electrical Activity in Adults from the Western Cape Region of South Africa

**DOI:** 10.3390/jcm10184112

**Published:** 2021-09-12

**Authors:** Cassidy Williams, Festus M. Kamau, Frans Everson, Boipelo Kgokane, Patrick De Boever, Nandu Goswami, Ingrid Webster, Hans Strijdom

**Affiliations:** 1 Centre for Cardio-Metabolic Research in Africa, Division of Medical Physiology, Faculty of Medicine and Health Sciences, Stellenbosch University, Cape Town 8000, South Africa; cassidywilliams70@gmail.com (C.W.); franseverson@gmail.com (F.E.); 19967519@sun.ac.za (B.K.); iwebster@sun.ac.za (I.W.); jgstr@sun.ac.za (H.S.); 2 Department of Biology, University of Antwerp, 2610 Wilrijk, Belgium; patrick.deboever@uantwerpen.be; 3 Centre for Environmental Sciences, Hasselt University, 3590 Diepenbeek, Belgium; 4 Division of Physiology, Otto Loewi Research Center of Vascular Biology, Immunity and Inflammation, Medical University of Graz, 8036 Graz, Austria; nandu.goswami@medunigraz.at

**Keywords:** HIV/AIDS, antiretroviral therapy, electrocardiography, cardiovascular disease, cardiometabolic risk

## Abstract

Cardiovascular-related complications are on the rise in people with HIV/AIDS (PWH); however, the relationship among HIV and antiretroviral therapy (ART)-related parameters, cardiovascular risk, and cardiac electrical activity in PWH remain poorly studied, especially in sub-Saharan African populations. We investigated whether HIV and ART are associated with cardiometabolic and cardiac electrical activity in PWH from Worcester in the Western Cape Province, South Africa. This was a cross-sectional study with HIV-negative (HIV−, *n* = 24) and HIV-positive on ART (HIV+/ART+, *n* = 63) participants. We obtained demographic, lifestyle, and medical history data and performed anthropometric, clinical assessments, and blood/urine biochemistry. We performed multiple stepwise linear regression analyses to determine independent associations among HIV, ART, cardiometabolic, and electrocardiographic (ECG) variables. HIV+/ART+ independently associated with a lower body mass index (*p* = 0.004), elevated gamma-glutamyl transferase levels (β: 0.333 (0.130–0.573); *p* = 0.002), and elevated alanine aminotransferase levels (β: 0.427 (0.224–0.629); *p* < 0.001) compared to HIV−. Use of second-line ART was positively associated with high-sensitivity C-reactive protein (*p* = 0.002). Although ECG parameters did not differ between HIV− and HIV+/ART+, viral load positively associated with *p*-wave duration (0.306 (0.018–0.594); *p* = 0.038), and longer HIV duration (≥5 years) with ST-interval (0.270 (0.003–0.537); *p* = 0.047) after adjusting for confounding factors. Our findings suggest that HIV and ART are associated with mixed effects on this population’s cardiometabolic profile and cardiac electrical activity, underpinning the importance of cardiovascular risk monitoring in PWH.

## 1. Introduction

HIV/AIDS remains a global health burden, affecting over 38 million globally and over 26 million people from sub-Saharan Africa (SSA) [1]. Antiretroviral therapy (ART) has superbly improved the prognosis of people with HIV/AIDS (PWH). However, HIV- and ART-associated cardiometabolic comorbidities and cardiovascular disease (CVD) complications are rising [2]. By 2030, 73% of PWH will be over 50 years of age, and more than 75% of those individuals will present with a form of CVD [3]. The pathophysiology of CVD in PWH is multifactorial. Factors such as the HI-virus, ART-associated side effects, traditional cardiovascular risk factors, and combinations thereof are culprits [4]. Evidence, mostly from developed countries, shows that HIV-positive (HIV+) individuals with or without ART are at an increased risk of developing cardiac complications, including dysregulation of the cardiac electrical activity [5]. Concomitantly, metabolic dysregulation and liver disease are commonly reported in PWH, and a link to CVD has been established [6].

Cardiovascular complications are of great concern in South Africa as the country hosts the largest HIV population and government-sponsored ART programme globally. Despite this concern, the cardiovascular health of PWH in South Africa is infrequently monitored and poorly researched. As the HIV+ population in South Africa becomes more ART experienced, the cardiac effects of HIV and ART warrant urgent research attention to prevent the overburdening of already limited health care resources. Electrocardiography is valuable and reliable in cardiovascular assessment and an essential tool for investigating arrhythmias and various cardiac disorders [5,7]. Evidence from a study executed in Nigeria shows that electrocardiogram (ECG) abnormalities are more prevalent among PWH with and without ART than HIV-negative (HIV−) individuals [8]. Another study reported that ECG abnormalities are common in Indian PWH who present without CVD symptoms [9], showing the possibility to identify individuals with sub-clinical electrical-mechanical cardiac changes before overt CVD manifestations.

Considering the paucity of data from the SSA region, this cross-sectional study investigates, for the first time, whether HIV and ART are associated with cardiometabolic and ECG variables in a study population from South Africa. In particular, we determined the cardiometabolic risk factors associated with HIV and ART, and analysed ECGs to determine whether these risk factors are associated with atrial/ventricular ECG alterations.

## 2. Methods

### 2.1. Study Design, Ethics, and Demographic Information

This current cross-sectional study was conducted as a sub-study embedded in a multi-site longitudinal parent study called EndoAfrica [10]. Ethics clearance was granted by the Stellenbosch Health Research Ethics Committee (ethics reference number: N19/02/029). Signed informed consent was obtained from all volunteers before participating in any study activity. Participants who presented with health concerns such as uncontrolled blood pressure, elevated blood glucose, or lipid levels in the study were promptly referred for relevant clinical intervention according to the Department of Health guidelines.

The study population consisted of participants from the Worcester community (Western Cape Province of South Africa) who presented for their 36-month follow-up visit as part of the EndoAfrica parent study. The study population comprised of HIV+ ART-exposed patients (HIV+/ART+, first-line ART regimen: tenofovir disoproxil fumarate (TDF), emtricitabine, and efavirenz or second-line lopinavir/ritonavir-based regimen (switched because of either virological, immunological or clinical failure of first-line regimen) and HIV− participants. Overall, HIV+ and HIV− participants were mainly recruited at health care clinics where they presented for routine medical care. We ensured that HIV− participants were recruited from the same community where the HIV+ participants live. We conducted home visits with help from local community health workers.

Participants younger than 18 years of age, pregnant women, women three months or less post-partum, participants with active (or on treatment for) tuberculosis, hepatitis B or C and severely sick individuals (AIDS stage 4) were excluded from the study.

Demographic, lifestyle, past medical history and socioeconomic data were collected from the participants by a qualified research nurse. Demographic information included age (years) and sex (male or female). Self-reported total monthly household income (≥/< R5000/month, ZAR) was included in this study as a marker of socioeconomic status. This figure represents the upper-bound poverty line per person per month (1227 ZAR) in an average South African household with four inhabitants, according to 2019 statistics South Africa [11]. Lifestyle information included current smoking status (Yes/No) and alcohol consumption (Yes/No and quantity) within the last year.

### 2.2. Anthropometric Measurements and Biochemical Analysis

A qualified research nurse conducted anthropometric measurements, urine collection, and phlebotomy. Anthropometric measurements were with standardised protocols [12]. They included height (cm), measured with a stadiometer, weight (kg), using an electronic scale, and waist and hip circumferences (cm) measured with a measuring tape. Body mass index (BMI, kg/m^2^) and waist-to-hip ratio were calculated. Systolic blood pressure (SBP, mmHg) and diastolic blood pressure (DBP, mmHg) were measured thrice at five-minute intervals using the Omron M6 automatic digital blood pressure monitor (Omron Healthcare, Kyoto, Japan) around the left brachium. Subsequently, the average value was calculated.

Study participants fasted from 10:00 pm the night before clinical assessments. Participants with unknown HIV status were tested for HIV using a rapid HIV test (SD Bioline HIV 1/2 3.0 immunochromatographic kit; Standard Diagnostics, Republic of Korea) to determine their HIV status. Urine and fasting blood samples were collected and sent to the National Health Laboratory Service (NHLS) for biochemical analyses using standard laboratory techniques. Plasma lipid profiles (total cholesterol (TC), high-density lipoprotein cholesterol (HDL-C), low-density lipoprotein cholesterol (LDL-C), and triglyceride levels (TG), mmol/L) were determined using chemiluminescence methodology (cobas^®^ 301/501 analyser, Roche/Hitachi cobas^®^ c systems, Basel, Switzerland). Levels of liver enzymes (gamma-glutamyl transferase (GGT, U/L) and alanine aminotransferase (ALT, U/L)) were determined via an enzymatic chemiluminescence methodology on a cobas^®^ 311/501 analyser, (Roche/Hitachi cobas^®^ c systems, Basel, Switzerland). Levels of high-sensitivity C-reactive protein (hsCRP, mg/L) were obtained via an IMMAGE^®^ Immunochemistry Systems and Calibrator 5 Plus assay kit (Beckman Coulter, Inc., CA, USA). Fasting glucose levels (mmol/L) and glycated haemoglobin (HbA1C, Hb%) were determined using chemiluminescence methodology (hemolysate application on a cobas^®^ 311/501 analyser, Roche/Hitachi cobas^®^ c systems, Basel, Switzerland). Haemoglobin (Hb, g/dL) levels were obtained using a chemiluminescence method (whole blood application on a cobas^®^ 311/501 analyser, Roche/Hitachi cobas^®^ c systems, Basel, Switzerland).

Urine samples were analysed to determine microalbuminuria (mg/L) and creatinine (µmol/L) using an enzymatic chemiluminescence method on cobas^®^ 501/502 and cobas^®^ 311/501 analysers, (Roche/Hitachi cobas^®^ c systems, Basel, Switzerland) respectively and albumin-to-creatinine ratio (ACR) (mg/mmol) was computed.

In the HIV+ participants, immuno-virological and other HIV− and ART-related data were collected. The levels of cluster of differentiation four (CD4)+ T cell count and viral load (VL) were determined by flow cytometry (FC 500 MPL) with MXP software (Beckman Coulter, Brea, CA, US) and COBAS^®^ AmpliPrep/COBAS^®^ TaqMan^®^ HIV-1 Test, v2.0 (Roche Diagnostics Ltd, Burgess Hill, UK), respectively. The duration of HIV infection (referred to as HIV duration in the results section and defined as < or ≥5 years), type of ART regimen (first-line or second-line), and duration on ART (referred to as ART duration in the results section and quantified as the number of weeks since ART commenced) were documented.

### 2.3. ECG Data Collection and Analysis

Single-channel ECGs were digitally recorded using the OMRON HeartScan HCG-801 ECG monitor (Omron Healthcare, Australia), a user-friendly and non-invasive device ideal for field studies [13]. We recorded the resting ECG for 30 seconds with participants in the supine position. Subsequent ECG analyses of resting heart rate (RHR), atrial, and ventricular cardiac electrical activity were performed using OMRON ECG Viewer 2.0 software (OMRON Healthcare, Australia). Features that reflect atrial electrical activity include P wave, PR segment, PR interval, and ventricular electrical activity features include QRS complex, ST segment, ST interval, T wave, and QT interval [14].

### 2.4. Statistical Analysis

All statistical analyses were performed using IBM^®^ SPSS^®^ software (version 25, New York, NY, USA). The data distribution for each variable was evaluated (Shapiro–Wilk tests, evaluating data histograms and Q-Q plots). Population characteristics are presented as mean ± standard deviation (SD) and median (range: minimum to maximum) for parametric and non-parametric continuous variables, respectively. Categorical variables are presented as the sample size (*n*, % of the study group). Independent sample t-tests or Mann–Whitney U tests were used to compare data between the two groups. Pearson Chi-squared tests were performed to compare categorical data, and Spearman correlations were performed to evaluate the relationship between continuous variables.

Independent associations between independent and dependent variables were determined using multiple stepwise linear regression analyses. A stepwise regression approach was done because of the substantial number of variables in the study (only significant confounders were included in each model). Variables with a skewed data distribution were log_10_^-^ transformed for regression analyses. Two models were applied. Variables considered in model A included: age (years), sex (male/female), active smoking (yes/no), alcohol consumption (yes/no), income level (</≥ R5000/month), BMI, and liver function enzymes (GGT, ALT). Model A was applied to all cardiometabolic variables. Model B considered model A confounders with SBP, DBP, TC, HDL-C, LDL-C, TG, fasting glucose, HbA1c, Hb, haematocrit, and ACR additionally considered. Model B was applied to all ECG variables. Additional cardiometabolic parameters were included in model B to elucidate possible associations between cardiometabolic and ECG variables.

When testing for associations with HIV-related variables, the regression models additionally included HIV and ART characteristics markers, *viz.* HIV-status (yes/no) for the total study population (HIV− and HIV+/ART+ groups combined), and for the HIV+/ART+ group only, VL, CD4+ T cell count, HIV duration (</≥ 5 years), ART duration (weeks), and ART-type (first- or second-line). 

Results for regression analyses are reported as standardised β coefficient (95% CI), *p*-value with R^2^, and adjusted R^2^. Full data tables for regression results are available in the supplementary material (independent associations with: body composition (Table S1), blood pressure (Table S2), lipid levels (Table S3), glucose levels (Table S4), liver enzyme levels (Table S5), haemoglobin and haematocrit levels (Table S6), kidney function (Table S7), inflammation (hsCRP, Table S8), RHR and RR interval (Table S9), atrial electrical activity (Table S10), and ventricular electrical activity (Table S11)). A summary of all findings is included as a figure (Figure S1). A *p*-value < 0.05 was considered significant for all statistical analyses.

## 3. Results

### 3.1. Population Characteristics

The study population (*n* = 63 HIV+/ART+ and *n* = 24 HIV−) was relatively young (22 to 59 years) and mostly women (60%). Significantly, more HIV+/ART+ participants were from households with a total household income of <R5000 per month compared to HIV− participants (*p* = 0.001). HIV+/ART+ presented with significantly lower body composition (weight, BMI, waist and hip circumference) compared to HIV−. 

Liver enzymes, GGT, and ALT were significantly higher and clinically elevated (GGT: ≥60 U/L for males and ≥40 U/L for females; ALT: ≥30 U/L for males and ≥19 U/L for females [15,16] in HIV+/ART+ compared to HIV− (*p* = 0.002 respectively). Although there were no significant differences in the mean hsCRP levels between the two groups, both groups presented with mean hsCRP levels above the upper limit (>3 mg/L). This cut-off value is associated with increased cardiovascular risk, as previously described by Ridker et al. [17]. Population characteristics are summarised below (Table 1).

### 3.2. HIV and ART Characteristics

Median VL and mean CD4+ T cell counts were within acceptable WHO cut-off values (<1000 mRNA copies/mL and >250 cells/mm^3^) [18]. There were no significant differences in VL and CD4+ T cell count levels between the two infection duration categories: <5 years and ≥5 years. However, HIV+/ART+ participants using second-line ART presented with a significantly higher VL than those using first-line ART. Use of second-line ART was associated with a significantly lower CD4+ T cell count than first-line ART. Duration of HIV (≥5 years) was associated with a longer ART duration and use of second-line ART (Table 2).

VL and CD4+ T cell count were inversely correlated (Spearman correlation: *r* = −0.465, *p* < 0.001). VL and CD4+ T cell count were not significantly associated with ART duration (Spearman correlation: *r* = −0.001, *p* > 0.05 and *r* = −0.080, *p* > 0.05). ART-type was not significantly associated with HIV duration (Pearson Chi-square: 0891, *p* = 0.345).

To determine whether independent associations between markers of HIV and ART (VL, HIV duration, ART duration, ART-type, CD4+ T cell count) and other population characteristics (dependent variable: household income, alcohol consumption, smoking and sex) exist, multiple forward binary logistic regression analysis was applied (Model: age, gender, smoking, alcohol consumption, income, VL, HIV duration, ART duration, ART-type, CD4+ T cell count). An HIV duration of ≥ 5 years was significantly associated with a lower total household income (B: −2.234, odds ratio: 0.107, (95% CI: 0.031−0.554), *p* = 0.008). 

### 3.3. Analysis of ECG Characteristics in HIV+/ART+ and HIV− Participants

We observed no significant differences in any of the ECG parameters between the study groups (Table 3). 

### 3.4. Associations between HIV-Related and Cardiometabolic/ECG Variables

In the total study population, HIV+/ART+ was inversely associated with BMI and positively associated with the liver enzyme levels (GGT and ALT) (Table 4). In the HIV+/ART+ group, the use of second-line ART was inversely associated with body composition (BMI and hip circumference), ALT and Hb, and positively associated with hsCRP. For detailed data tables on body composition and liver enzyme levels, refer to Supplementary Tables S1 and S5, respectively. VL was inversely associated with blood pressure (SBP and DBP) and the liver enzyme GGT, while a positive association between VL and atrial electrical activity (P wave duration) was observed. HIV duration (≥5 years) was furthermore inversely associated with hsCRP and positively associated with ventricular electrical activity (ST interval) (Table 4). For complete data tables on inflammatory marker hsCRP and ventricular electrical activity, refer to Supplementary Tables S8 and S11, respectively.

### 3.5. Associations between Cardio-Metabolic and ECG Variables in the HIV + ART + Group

The liver enzyme, ALT, was positively associated with the P wave duration and BMI was positively associated with the PR interval (Table 5). For complete data on associations with atrial electrical activity, refer to Supplementary Table S10. Hb levels were positively associated with ventricular electrical activity (ST interval, T wave duration and QT interval). Systolic blood pressure was positively associated with the QRS complex and T wave duration (Table 5). For complete data on associations with ventricular electrical activity, refer to Supplementary Table S11. 

## 4. Discussion

The current cross-sectional study investigates whether HIV and ART are associated with cardiometabolic and ECG variables in a study population from Worcester in South Africa. The study aimed to determine whether cardiometabolic variables are associated with atrial and ventricular ECG alterations in ART-experienced PWH. Overall, we found that HIV and ART are independently associated with several cardiometabolic and ECG variable outcomes in this study population. We observed higher liver enzyme levels (ALT and GGT) in HIV+/ART+ compared to HIV− participants. HIV+/ART+ status (particularly second-line ART) associated positively with ALT and GGT, and negatively associated with BMI, SBP, DBP, and total cholesterol. Treatment with second-line ART was positively associated with systemic inflammation (hsCRP) and negatively associated with ALT. Whereas VL and ≥5 years’ duration of HIV-infection were positively associated with atrial (P-wave duration) and ventricular (ST interval) electrical activity, respectively, VL associated negatively with liver enzymes. In HIV+/ART+ participants, several cardiometabolic variables showed positive associations with measurements of atrial and ventricular electrical activity, with ALT, BMI, Hb, and SBP showing significance (*p* ≤ 0.01).

In our study, ECG variables did not differ significantly between HIV− and HIV+/ART+. This is in contrast to a previous study from China that showed a high prevalence of sinus tachycardia (heart rate > 100 bpm), ST segment elevation and left ventricular hypertrophy in PWH [20]. A key difference with our study is that 38% of their study population was between the ages of 45 and 75 years and >60% reported comorbidities. A cross-sectional study in Nigeria reported a higher prevalence of ECG abnormalities (T wave inversion and a prolonged QT interval) in HIV+/ART+ participants compared to ART-naïve and HIV− participants [8]. In Cameroon, ECG findings among PWH included abnormal repolarisation, sinus tachycardia, left ventricular hypertrophy, conduction anomalies, and arrhythmias [21]. The differences could be because of recruitment of participants with symptomatic CVD, differences in ART regimens and the more predominant HIV-2 subtype in Western Africa. Our population was relatively young, and we excluded individuals with severe illnesses. Such differences reflect the diversity in the direct and indirect interactions between CVD risk factors and markers of HIV that may affect the electrical activity of the heart in PWH.

After adjusting for confounding factors, we observed a positive association between VL and P wave duration (representing atrial depolarisation). Furthermore, 11% of HIV+/ART+ participants in our study presented with abnormal P wave durations. These findings support Okoye and Anyabolu [22], who showed that 13% of the HIV+ participants presented with abnormal P waves. They also reported that abnormalities in atrial and ventricular electrical activities were more prevalent in HIV+ participants with a lower BMI. They found that the mean PR interval was significantly higher in their HIV+ participants compared to HIV− controls; however, HIV+ participants were treatment naïve, which may account for discrepancies [22]. PR interval was associated with body composition (BMI) in the present study. Previous evidence has shown that HIV+ individuals presented with prolonged atrial electromechanical delays, which may place these individuals at risk of atrial arrhythmias [23]. These findings suggest a plausible relationship between body composition and atrial activity in PWH, but they warrant more extensive investigations to determine the underlying mechanisms.

Longer HIV duration (≥5 years) was positively associated with ST interval (representing ventricular repolarisation) in the current study. Our findings support the results of a study by Ding et al. [24], who showed that ST/T wave abnormalities were positively associated with HIV infection, ACR, Hb, fasting glucose and longer duration of HIV infection (≥3 years). ST/T wave abnormalities have been associated with the risk of developing ventricular tachycardia, myocardial ischaemia, myocardial infarction, and abnormal ventricular repolarisation [25]. This finding, however, contradicts Ding et al. [24], who reported significant associations between blood pressure categories and ventricular abnormalities (ST/T abnormalities) in their HIV− group instead. Soliman et al. [26], reported that ST/T abnormalities, in particular, may be predictive of CVD in PWH and highlighted the prognostic value of ST/T changes in HIV+ patients. Ding et al. [24], observed that a long duration of HIV was the only HIV-specific factor for ST/T abnormalities suggesting a possible biological link. We found that BMI, ALT, and triglycerides were positively associated with altered atrial electrical activity. It is possible that atrial electrical changes precede overt cardiometabolic, atrial and ventricular mechanical derangements in PWH. These results suggest that prolonged HIV duration in the current study population may increase the risk of developing various cardiac complications. However, clear pathophysiology and early intervention strategies ought to be explored.

In our study, HIV+/ART+ was inversely associated with BMI compared to HIV−. Although the use of ART in PWH is often associated with the reversal of HIV-associated weight loss [27], ART was not significantly associated with an increase in body weight. In a pooled analysis of eight randomised control studies comprised of >5000 participants, the authors found that the rate of weight gain in PWH who started ART was most rapid during the initial 48 weeks, although 30% of these individuals recorded weight loss [28]. Furthermore, populations from a lower socioeconomic status may be disproportionately affected in terms of HIV infection risk; the corollary of this is impaired general wellbeing in these populations. It is possible that a higher socioeconomic status in HIV− participants compared to HIV+/ART+ participants will contribute to obesogenic dietary behaviours. Future longitudinal follow-up studies may be valuable in providing information about the temporal changes in body composition phenotypes in this specific population.

The lipid profiles did not significantly differ between HIV+/ART+ and HIV− participants and, were within acceptable clinical ranges. Dyslipidaemia as a result of primary HIV infection, as well as treatment with ART, is often reported [29]. Our findings support Riddler et al. [30], who opined that the use of ART results in a return to pre-HIV infection serum lipid levels. In contrast, Menanga et al. [21] reported ART-associated dyslipidaemia in 38% of their HIV+ study sample. The D:A:D study reported that use of ART regimens containing non-nucleoside reverse transcriptase inhibitors and protease inhibitors was associated with a higher prevalence of dyslipidaemia, but the examined cohort lived in high-income countries [31]. This may account for conflicting findings in the current study, as most HIV+/ART+ participants were mainly from a low socioeconomic status and did not use protease inhibitors.

GGT and ALT levels were significantly higher in HIV+/ART+ individuals compared to controls and positively associated with an HIV+ status in our study population. These findings support those of previous reports where ART-associated liver toxicity was observed [32,33]. A study in Namibia reported significant elevation in ALT levels specifically compared to grading criteria in PWH using first-line ART [34]. Kakako and Najim [35], showed that elevated ALT levels were associated with first-line and second-line ART. The first-line regimen in the present study contained efavirenz (EFV). Segamwenge and Bernard [36], reported severe EFV-associated hepatocellular injury following long-term EFV use. Previous studies have shown that GGT levels provide an accurate and sensitive biomarker for the prediction of CVD [37]. Manifestations of liver abnormalities, such as ART-associated fatty liver disease, play an important role in CVD development in PWH [38,39]. Therefore, our findings suggest that despite the development of newer, less toxic ART regimens [40], regular monitoring of liver function in study populations, such as in this sub-study, is still recommended.

Chronic inflammation in PWH, often a consequence of chronic immune activation, has been associated with cardiovascular risk [41,42,43]. Elevated levels of circulating hsCRP (>3 mg/L), a marker of systemic inflammation, has previously been associated with increased cardiovascular risk [17]. Hsue et al. [44], reported in their cross-sectional study that hsCRP levels were elevated among PWH compared to their HIV− counterparts. Our study does not support these findings. Despite no significant differences in hsCRP levels between the HIV+/ART and HIV− groups, the mean hsCRP levels in HIV− and HIV+/ART+ participants were two- to three-times higher than the upper cut-off level (3 mg/L); portending high cardiovascular risk in both groups. In the current study, hsCRP was positively associated with the use of second-line ART and inversely associated with duration of HIV infection. Cumulatively, the data suggests that viral suppression due to ART may not yield regulated inflammatory responses. In future studies, we should consider more inflammatory markers. Our results suggest we should place greater emphasis on exploring anti-inflammatory treatments used with ART.

Atrial and ventricular ECG parameters were not associated with lower CD4+ T cell count. This is in contrast to evidence from previous studies. Shen et al. [20], reported that a lower CD4+ T cell count was positively associated with sinus tachycardia. Sakthivadivel et al. [45], observed a significant association between ECG abnormalities and CD4+ T cell counts and subsequently reported that PWH with a CD4+ T cell count ≤ 350 cells/mm^3^ are more vulnerable to ECG changes compared to PWH with a CD4+ T cell count > 350 cells/mm^3^. In recent studies, CD4+ T cell count (≤350 cells/mm^3^) was an indicator of increased risk for CVD [7]. One explanation for the differences observed may be that the HIV+/ART+ participants in the present study, irrespective of ART regimen, recorded a mean CD4+ T cell count above the threshold for potential ECG abnormalities compared to previous studies. Therefore, through ART-associated viral suppression, immune system restoration occurs, as evidenced by high CD4+ T cells [46]. Immune restoration may be cardioprotective through a reversal of HIV-related effects on the electrical activity of the heart and cardiometabolism, but this ought to be investigated further.

## 5. Conclusions

The current study investigated whether HIV and ART were associated with cardiometabolic effects and markers of the electrical activity of the heart in a study population living in the Worcester area of the Western Cape Province, South Africa. Although HIV+/ART+ participants presented with a median BMI value that fell within the normal clinical range, and no evidence of dyslipidaemia, liver enzymes were significantly elevated compared to HIV− participants. Collectively, our results suggest that cardiometabolic parameters, as well as markers of HIV and ART, have diverse associations with the electrical activity of the heart in PWH using ART. The observations suggest an intricate interplay between HIV, ART, cardiometabolic risk factors, and cardiac electromechanical activity. Additionally, VL and HIV duration independently associated with risk for altered atrial and ventricular activity, possibly showing risk for early cardiac electromechanical abnormalities. Our findings underscore the need for early, regular assessment of cardiac risk in PWH, especially in populations similar to the ones under scrutiny. Finally, in the broader context of South Africa and the greater SSA region, more robust research is warranted to explore the mechanisms involved in the development of ECG abnormalities and the HIV-related and cardiometabolic factors that contribute to these abnormalities.

## 6. Study Limitations

Our study followed a cross-sectional approach and we consider this a limitation, as it does not allow for investigation of a progressive/retrogressive relationship between markers of HIV infection, cardiometabolic parameters and ECG variables. Follow-up assessments and data collection are currently underway. The study population was largely composed of female participants. Our results are thus more representative for women, although comprising a relatively small sample size. Future studies should consider a more equally distributed study population in terms of sex. It is important to note that although hidden or important relationships can be found in exploratory analyses that employ stepwise regression analyses, there is the possibility of spurious correlations being discovered. We consider this a limitation of the present study because we performed multiple stepwise linear regression analyses to determine independent associations among HIV, ART, cardiometabolic, and ECG variables. A study strength is that we collected these cardiovascular data in the Western Cape province of South Africa for the first time. Although single-channel ECGs were sufficient for analyses in the present study, 12-lead ECGs are recommended for future studies as it may be more sensitive in detecting ECG abnormalities. However, there is a trade-off and our single-channel ECG has clear logistical merits during the fieldwork. Despite the aforementioned limitations, the present study adds to the limited body of evidence on HIV and cardiovascular risk in PWH in South Africa and SSA.

## Figures and Tables

**Table 1:** Population characteristics.

Variable ^a^	HIV−	HIV+/ART+	*p*-Value
*n*/group	24	63	
Age, years	49 (28 to 59)	43 (22 to 59)	0.248
Sex, female, *n* (%)	17 (71%)	35 (56%)	0.195
Smoking status, yes, *n* (%)	11 (42%)	30 (48%)	0.585
Alcohol consumption, yes, *n* (%)	10 (46%)	33 (51%)	0.619
Household income/month			
<R5000/month, n (%)	10 (42%)	49 (78%)	0.001
≥R5000/month, n (%)	14 (58%)	14 (22%)	
Body mass index (BMI), kg/m^2^	27.7 (16.8 to 45.6)	22.1 (15.2 to 42.5)	0.023
BMI classification levels			
Underweight (<18.5 kg/m^2^), n (%)	1 (4%)	12 (19%))	
Normal weight (18.5 to < 25 kg/m^2^), n (%)	7 (29%)	30 (48%)	
Overweight (25 to < 30 kg/m^2^), n (%)	6 (25%)	11 (18%)	
Obese (>30 kg/m^2^), n (%)	10 (42%)	10 (16%)	
Waist, cm	100 (77 to 140)	90 (69 to 143)	0.024
Waist-to-hip ratio	0.97 (0.76 to 1.04)	0.97 (0.79 to 1.20)	0.849
Systolic blood pressure (SBP)	125 ± 17.5	118 ± 14.5	0.098
Diastolic blood pressure (DBP)	87 ± 12.7	83 ± 9.8	0.143
Lipid profile			
Total cholesterol (TC), mmol/L	4.39 (2.64 to 6.5)	4.46 (2.26 to 8.84)	0.636
High-density lipoprotein cholesterol (HDL-C), mmol/L	1.28 (0.74 to 2.13)	1.31 (0.57 to 5.36)	0.827
Low-density lipoprotein cholesterol (LDL-C), mmol/L	2.74 ± 0.95	2.44 ± 0.76	0.172
Triglycerides (TG), mmol/L	0.98 (0.43 to 3.77)	1.02 (0.35 to 6.25)	0.708
Glucose metabolism			
Fasting glucose level, mmol/L	4.85 (3.9 to 10.1)	4.7 (2.0 to 13.7)	0.461
Glycated haemoglobin (HbA1C), %	5.6 (5.1 to 11.0)	5.5 (4.4 to 10.8)	0.064
Haemoglobin (Hb), g/dL	13.9 (12.0 to 16.8)	13.8 (7.8 to 18.0)	0.436
Haematocrit, L/L	0.44 (0.37 to 0.53)	0.43 (0.31 to 0.58)	0.679
hsCRP, mg/L	7.9 ± 7.6	11.3 ± 15.6	0.191
Liver function			
Gamma-glutamyl transferase (GGT), U/L	28.5 (11.0 to 83.0)	41.0 (14.0 to 219)	0.002
Alanine aminotransferase (ALT), U/L	14.5 (6.0 to 33.0)	20.0 (7.0 to 44.0)	0.002
Kidney function			
Serum creatinine, μmol/L	63.1 ± 12.3	66.6 ± 14.32	0.266
Albuminuria, mg/L	6.95 (1.2 to 97.3)	12.5 (0.9 to 3570)	0.252
Albumin-to-creatinine ratio (ACR), mg/mmol	0.90 (0.30 to 5.50)	0.90 (0.20 to 305)	0.798

^a^ Data presented as median (range), mean ± standard deviation (SD) or *n* (% of study group) for *n* = 87 participants (HIV−: *n* = 24, HIV+/ART+: *n* = 63).

**Table 2:** VL, CD4+ T count, and ART duration categorised per HIV duration and ART regimen.

HIV Duration and ART Categories			*p*-Value
Viral load (VL)		50 (10 to 424,343)	
HIV duration	<5 years (15 (24%)) ≥5 years (48 (76%))	10 (10 to 424,353) 50 (10 to 387,891)	0.536
ART type	First-line (53 (84%)) Second-line (10 (16%))	50 (10 to 313,857) 10,274 (10 to 424,353)	0.009
CD4+ T cell count, cells/mm^3^		478 ± 14.5	
HIV duration	<5 years (15 (24%)) ≥5 years (48 (76%))	466 (69 to 889) 513 (66 to 893)	0.915
ART type	First-line (53 (84%)) Second-line (10 (16%))	532 (123 to 893) 181 (66 to 708)	0.003
ART duration (weeks)		249 (0 to 782)	
HIV duration	<5 years (15 (24%)) ≥5 years (48 (76%))	169 (12 to 247) 276 (12 to 782)	<0.001
ART type	First-line (53 (84%)) Second-line (10 (16%))	236 (12 to 604) 341 (166 to 610)	0.028

Data presented as median (range), mean ± standard deviation (SD) or *n* (% of study group, HIV+/ART+: *n* = 63).

**Table 3:** Comparison of ECG variables between HIV+/ART+ and HIV− participants.

Variable ^a^	Cut-Off Standards [19]	HIV−	HIV+/ART+	*p*-Value
Resting Heart Rate (RHR), bpm	60–100	67.7 ± 10.4	72.8 ± 14.1	0.070
RR interval, s	0.6–1.2	0.88 (0.68 to 1.20)	0.84 (0.56 to 1.32)	0.289
*p* wave duration, ms	80–120	83.3 (53.3 to 126.7)	80.0 (60.0 to 126.7)	0.093
PR interval, ms	120–200	157 (120 to 213)	140 (80 to 200)	0.075
PR segment, ms	50–120	64.7 ± 19.1	56.2 ± 19.3	0.070
QRS complex, ms	60–100	90.0 (80.0 to 106.6)	93.3 (73.3 to 120.0)	0.580
ST segment, ms	80–120	100 (80.0 to 147)	87 (53 to 160)	0.069
T wave duration, ms	≈160	177 (140 to 200)	167 (120 to 240)	0.675
ST interval, ms	300–320	290 (240 to 333)	293 (180 to 333)	0.810
QT interval, ms	400–420	400 (360 to 473)	400 (280 to 440)	0.131

^a^ Data presented as median (range), mean ± standard deviation (SD) for *n* = 87 participants (HIV−: *n* = 24, HIV+/ART+: *n* = 63).

**Table 4:** Significant associations between HIV and cardiometabolic variables.

Dependent Variable	Independent Variable	Standardised β (95% CI)	*p*-Value
Body composition ^a^			
BMI	HIV status (+)	−0.282 (−0.427 to −0.092)	0.004
	ART (second-line)	−0.239 (−0.461 to −0.017)	0.036
Hip circumference	ART (second-line)	−0.247 (−0.474 to −0.020)	0.034
Blood pressure ^a^			
SBP	Viral load	−0.279 (−0.540 to −0.018)	0.037
DBP	Viral load	−0.325 (−0.565 to −0.085)	0.009
Lipid levels ^a^			
TC	Viral load	−0.414 (−0.654 to −0.174)	0.001
Liver function ^a^			
GGT	HIV status (+)	0.333 (0.130 to 0.537)	0.002
	Viral load	−0.299 (−0.580 to −0.018)	0.038
ALT	HIV status (+)	0.427 (0.224 to 0.629)	<0.001
	ART (second-line)	−0.333 (−0.593 to −0.073)	0.013
Haemoglobin ^a^			
Hb	ART(second-line)	−0.373 (−0.649 to −0.097)	0.009
Inflammation ^a^			
hsCRP	ART (second-line)	0.510 (0.198 to 0.821)	0.002
	HIV duration (≥5 years)	−0.333 (−0.636 to −0.030)	0.032
Atrial Electrical Activity ^b^			
P wave duration	Viral load	0.306 (0.018 to 0.594)	0.038
Ventricular Electrical Activity ^b^			
ST interval	HIV duration (≥5 years)	0.270 (0.003 to 0.537)	0.047

^a^ Model A (variables considered): age, sex, active smoking, alcohol consumption, monthly household income, BMI, GGT and. ALT. ^b^ Model B: (variables considered): variables included in model A, SBP, DBP, TC, HDL, LDL, TG, fasting glucose, HbA1c, Hb, haematocrit and ACR.

**Table 5:** Significant associations between cardiometabolic parameters and atrial/ventricular electrical activity in the HIV+/ART+ study population.

Dependent Variable	Independent Variable	Standardised β (95% CI)	*p*-Value
Atrial electrical activity			
P wave duration	ALT	0.538 (0.226 to 0.851)	0.001
PR interval	BMI	0.407 (0.103 to 0.710)	0.010
PR segment	Triglycerides	0.373 (0.049 to 0.697)	0.025
Ventricular electrical activity			
QRS complex	SBP	0.367 (0.061 to 0.673)	0.020
ST segment	ACR	0.222 (0.006 to 0.439)	0.044
ST interval	Hb	0.441 (0.197 to 0.684)	0.001
T wave duration	SBP	0.487 (0.214 to 0.761)	0.001
	Hb	0.303 (0.074 to 0.531)	0.011
	Fasting glucose	0.244 (0.010 to 0.477)	0.041
QT interval	Hb	0.258 (0.030 to 0.486)	0.027
	Alcohol consumption (yes)	0.256 (0.021 to 0.490	0.033

Model B (variables considered): variables included in model A, SBP, DBP, TC, HDL, LDL, TG, fasting glucose, HbA1c, Hb, haematocrit, and ACR.

## Data Availability

The data presented in this study are available in the supplementary material.

## Supplementary Materials

The following are available online at https://www.mdpi.com/article/10.3390/jcm10184112/s1, Figure S1: Summary of regression results; Table S1: Independent associations with body composition; Table S2: Independent associations with blood pressure; Table S3:Independent associations with lipid levels; Table S4: Independent associations with glucose levels; Table S5: Independent associations with liver enzyme levels; Table S6: Independent associations with haemoglobin and haematocrit levels; Table S7: Independent associations with kidney function; Table S8: Independent associations with inflammation (hsCRP); Table S9: Independent associations with RHR and RR interval; Table S10: Independent associations with atrial electrical activity; Table S11: Independent associations with ventricular electrical activity.
