# Supplementary material for: HIV and Antiretroviral Therapy Are Independently Associated with Cardiometabolic Variables and Cardiac Electrical Activity in Adults from the Western Cape Region of South Africa
Source: J Clin Med. 2021 Sep 12;10(18):4112. doi: 10.3390/jcm10184112 (PMC8466610; doi:10.3390/jcm10184112)
Supplement: Supplementary file 1 [file jcm-10-04112-s001.zip › jcm-1360164-supplementary.pdf]

Supplementary Data: HIV and antiretroviral therapy are independently associated with cardiometabolic variables and cardiac electrical activity in adults from the Western Cape region of South Africa

Supplementary Table S1: Independent associations with body composition.

	**Independent variable**	**Standardised β (95%CI)**	***p*-value**
		**Weight**	
Total study population[a]	Income (> R5000)	0.363 (0.168 to 0.558)	< 0.001
	Smoking (yes)	-0.230 (-0.425 to -0.036)	0.021
HIV+/ART+[b]	Income (> R5000)	0.262 (0.023 to 0.502)	0.032
	Smoking (yes)	-0.316 (-0.538 to -0.093)	0.006
		**BMI**	
Total study population[c]	Income (> R5000)	0.284 (0.105 to 0.463)	0.002
	Smoking (yes)	-0.253 (-0.423 to -0.083)	0.004
	Sex (women)	0.282 (0.111 to 0.453)	0.002
	HIV status (+)	-0.282 (-0.427 to -0.092)	0.004
	ALT	0.258 (0.077 to 0.440)	0.006
HIV+/ART+[d]	Smoking (yes)	-0.443 (-0.655 to -0.230)	0.000
	Income (> R5000)	0.256 (0.026 to 0.485)	0.030
	ART (2nd-line)	-0.239 (-0.461 to -0.017)	0.036
		**Hip circumference**	
Total study population[e]	Sex (women)	0.363 (0.161 to 0.512)	< 0.001
	Income (> R5000)	0.316 (0.059 to 0.427)	0.001
	Smoking (yes)	-0.292 (-0.459 to -0.110)	0.001
HIV+/ART+[f]	Smoking (yes)	-0.485 (-0.703 to -0.266)	< 0.001
	ART (2nd-line)	-0.247 (-0.474 to -0.020)	0.034
		**Waist circumference**	
Total study population[g]	Income (> R5000)	0.336 (0.150 to 0.521)	0.001
	Smoking (yes)	-0.258 (-0.447 to -0.069)	0.008
	Sex (women)	0.211 (0.025 to 0.397)	0.027
	Age	0.186 (< 0.001 to 0.372)	0.049
HIV+/ART+[h]	Smoking (yes)	-0.401 (-0.622 to -0.179)	0.001
	Income (> R5000)	0.251 (0.012 to 0.490)	0.040
		**Waist-to-hip ratio**	
Total study population[i]	Sex (women)	-0.288 (0.494 to -0.081)	0.007
HIV+/ART+	No association		

Model A (variables considered): age, sex, active smoking, alcohol consumption, income level, BMI, GGT and ALT. Weight: [a] R^2 = 0.203, adjusted R^2 =0.184. [b] R^2 = 0.398, adjusted R^2 = 0.206. BMI: [c] R^2 = 0.444, adjusted R^2 =0.410. [d] R^2 = 0.350, adjusted R^2 = 0.313. Hip: [e] R^2 = 0.372, adjusted R^2 = 0.350. [f] R^2 = 0.297, adjusted R^2 = 0.271. Waist: [g] R^2 = 0.299, adjusted R^2 = 0.265. [h] R^2 = 0.398, adjusted R^2 = 0.260. Waist-to-hip ratio: [i] R^2 = 0.083, adjusted R^2 = 0.072.

Supplementary Table S2: Independent associations with blood pressure.

	Independent variable	Standardised β (95%CI)	*p*-value
	**SBP**		
Total study population[a]	BMI	0.228 (0.024 to 0.432)	0.029
HIV+/ART+[b]	Viral load	-0.279 (-0.540 to -0.018)	0.037
	**DBP**		
Total study population[c]	BMI	0.235 (0.029 to 0.441)	0.026
HIV+/ART+[d]	Viral load	-0.325 (-0.565 to -0.085)	0.009
	Sex (women)	0.223 (0.004 to 0.443)	0.046

Model A (variables considered): age, sex, active smoking, alcohol consumption, income level, BMI, GGT and ALT. SBP: [a] $R^2 = 0.117$, adjusted $R^2 = 0.096$. [b] $R^2 = 0.168$, adjusted $R^2 = 0.137$. DBP: [c] $R^2 = 0.104$, adjusted $R^2 = 0.083$. [d] $R^2 = 0.235$, adjusted $R^2 = 0.191$.

Supplementary Table S3: Independent associations with lipid levels.

	Independent variable	Standardised β (95%CI)	*p*-value
	**Total cholesterol**		
Total study population[a]	Sex (women)	0.334 (0.138 to 0.530)	0.001
	GGT	0.299 (0.102 to 0.495)	0.003
	Income (> R5000)	0.198 (0.003 to 0.393)	0.047
HIV+/ART+[b]	Viral load	-0.414 (-0.654 to -0.174)	0.001
	Sex (women)	0.380 (0.149 to 0.611)	0.002
	Smoking (yes)	0.331 (0.096 to 0.566)	0.007
	**High-density lipoprotein**		
Total study population[c]	Sex (women)	0.458 (0.265 to 0.651)	< 0.001
	BMI	-0.395 (-0.599 to -0.191)	< 0.001
	Smoking (yes)	-0.291 (-0.486 to -0.095)	0.004
	GGT	0.253 (0.070 to 0.435)	0.007
HIV+/ART+[d]	Sex (women)	0.644 (0.410 to 0.877)	< 0.001
	BMI	-0.513 (-0.772 to -0.253)	< 0.001
	ALT	0.415 (0.167 to 0.662)	0.001
	**Low-density lipoprotein**		
Total study population[e]	Income (> R5000)	0.293 (0.086 to 0.499)	0.006
	Sex (women)	0.232 (0.028 to 0.437)	0.027
HIV+/ART+	No associations		
	**Triglycerides**		
Total study population[f]	Sex (women)	-0.204 (-0.395 to -0.012)	0.037
	Age	0.300 (0.110 to 0.489)	0.002
	Income (> R5000)	0.275 (0.086 to 0.465)	0.005
	GGT	0.229 (0.039 to 0.419)	0.019
	Smoking (yes)	0.218 (0.026 to 0.411)	0.027
HIV+/ART+[g]	Sex (women)	-0.335 (-0.582 to -0.088)	0.009
	Income (> R5000)	0.281 (0.010 to 0.551)	0.042

Model A (variables considered): age, sex, active smoking, alcohol consumption, income level, BMI, GGT and ALT. TC: [a] $R^2 = 0.207$, adjusted $R^2 = 0.178$. [b] $R^2 = 0.320$, adjusted $R^2 = 0.281$. HDL: [c] $R^2 = 0.322$, adjusted $R^2 = 0.288$. [d] $R^2 = 0.423$, adjusted $R^2 = 0.390$. LDL: [e] $R^2 = 0.144$, adjusted $R^2 = 0.124$. Triglycerides: [f] $R^2 = 0.282$, adjusted $R^2 = 0.238$. [g] $R^2 = 0.190$, adjusted $R^2 = 0.160$.

Supplementary Table S4: Independent associations with glucose levels.

	Independent variable	Standardised β (95%CI)	*p*-value
	**Fasting glucose**		
Total study population[a]	BMI	0.285 (0.088 to 0.481)	0.005
	Age	0.249 (0.054 to 0.443)	0.013
	ALT	0.228 (0.033 to 0.424)	0.022
HIV+/ART+[b]	ALT	0.382 (0.120 to 0.645)	0.005
	BMI	0.330 (0.058 to 0.603)	0.018
	**HbA1c**		
Total study population[c]	BMI	0.404 (0.207 to 0.602)	< 0.001
	Sex (women)	-0.310 (-0.506 to -0.115)	0.002
	Age	0.240 (0.053 to 0.427)	0.012
HIV+/ART+[d]	ALT	0.289 (0.062 to 0.517)	0.014
	Age	0.272 (0.049 to 0.494)	0.018

Model A (variables considered): age, sex, active smoking, alcohol consumption, income level, BMI, GGT and ALT. Fasting glucose: [a] R^2 = 0.227, adjusted R^2 = 0.199. [b] R^2 = 0.275, adjusted R^2 = 0.247. HbA1c: [c] R^2 = 0.294, adjusted R^2 = 0.260. [d] R^2 = 0.185, adjusted R^2 = 0.155.

Supplementary Table S5: Independent associations with liver enzyme levels.

	Independent variable	Standardised β (95%CI)	*p*-value
	**GGT**		
Total study population[a]	HIV status (+)	0.333 (0.130 to 0.537)	0.002
HIV+/ART+[b]	Viral load	-0.299 (-0.580 to -0.018)	0.038
	**ALT**		
Total study population[c]	HIV status (+)	0.427 (0.224 to 0.629)	< 0.001
	BMI	0.385 (0.174 to 0.597)	< 0.001
	Sex (women)	-0.247 (-0.447 to -0.047)	0.016
HIV+/ART+[d]	ART (2nd-line)	-0.333 (-0.593 to -0.073)	0.013

Model A (variables considered): age, sex, active smoking, alcohol consumption, income level, BMI, GGT and ALT. GGT: [a] R^2 = 0.111, adjusted R^2 = 0.109. [b] R^2 = 0.078, adjusted R^2 = 0.061. ALT: [c] R^2 = 0.245, adjusted R^2 = 0.218. [d] R^2 = 0.109, adjusted R^2 = 0.092.

Supplementary Table S6: Independent associations with haemoglobin and haematocrit levels.

	Independent variable	Standardised β (95%CI)	*p*-value
	**Haemoglobin**		
Total study population[a]	Sex (women)	-0.264 (-0.473 to -0.056)	0.014
HIV+/ART+[b]	ART (2nd-line)	-0.373 (-0.649 to -0.097)	0.009
	Smoking (yes)	0.302 (0.036 to 0.569)	0.027
	**Haematocrit**		
Total study population[c]	Smoking (yes)	0.305 (0.103 to 0.506)	0.003
	Sex (women)	-0.232 (-0.433 to -0.031)	0.024
HIV+/ART+[d]	Smoking (yes)	0.488 (0.229 to 0.747)	< 0.001
	ALT	0.336 (0.069 to 0.602)	0.015

Model A (variables considered): age, sex, active smoking, alcohol consumption, income level, BMI, GGT and ALT. **Haemoglobin:** [a] R^2 = 0.070, adjusted R^2 = 0.059. [b] R^2 = 0.214, adjusted R^2 = 0.184. **Haematocrit:** [c] R^2 = 0.170, adjusted R^2 = 0.151. [d] R^2 = 0.273, adjusted R^2 = 0.246.

Supplementary Table S7: Independent associations with kidney function.

	Independent variable	Standardised β (95%CI)	*p*-value
	**Serum creatinine**		
Total study population[a]	Sex (women)	-0.577 (-0.750 to -0.404)	< 0.001
	Smoking (yes)	-0.204 (-0.377 to -0.030)	0.022
HIV+/ART+[b]	Sex (women)	-0.446 (-0.652 to -0.225)	< 0.001
	Alcohol consumption (yes)	-0.265 (-0.496 to -0.060)	0.022
	**Albumin-to-creatinine ratio**		
Total study population[c]	Age	0.223 (0.012 to 0.433)	0.038
HIV+/ART+[d]	Age	0.448 (0.162 to 0.735)	0.003

Model A (variables considered): age, sex, active smoking, alcohol consumption, income level, BMI, GGT and ALT. Serum creatinine: [a] R^2 = 0.387, adjusted R^2 = 0.365. [b] R^2 = 0.358, adjusted R^2 = 0.321. ACR: [c] R^2 = 0.050, adjusted R^2 = 0.039. [d] R^2 = 0.157, adjusted R^2 = 0.141.

Supplementary Table S8: Independent associations with inflammation (hsCRP).

	Independent variable	Standardised β (95%CI)	*p*-value
Total study population[a]	ALT	-0.269 (-0.419 to -0.046)	0.019
HIV+/ART+[b]	ART (2nd-line)	0.510 (0.198 to 0.821)	0.002
	HIV duration (> 5 years)	-0.333 (-0.636 to -0.030)	0.032
	Smoking (yes)	0.298 (0.005 to 0.590)	0.046

Model A (variables considered): age, sex, active smoking, alcohol consumption, income level, BMI, GGT and ALT. hsCRP: [a] R^2 = 0.068, adjusted R^2 = 0.056. [b] R^2 = 0.261, adjusted R^2 = 0.216.

Supplementary Table S9: Independent associations with RHR and RR interval.

	Independent variable	Standardised β (95%CI)	*p*-value
	**Resting heart rate**		
Total study population[a]	hsCRP	0.485 (0.304 to 0.666)	< 0.001
	GGT	0.304 (0.119 to 0.488)	0.002
HIV+/ART+[b]	hsCRP	0.460 (0.258 to 0.662)	< 0.001
	GGT	0.351 (0.116 to 0.587)	0.004
	**RR-interval**		
Total study population[c]	hsCRP	-0.424 (-0.609 to -0.239)	< 0.001
	GGT	-0.277 (-0.465 to -0.088)	0.005
HIV+/ART+[d]	hsCRP	-0.392 (-0.588 to -0.196)	< 0.001
	GGT	-0.306 (-0.535 to -0.078)	0.010

Model B (variables considered): variables included in model A, SBP, DBP, TC, HDL, LDL, TG, fasting glucose, HbA1c, haematocrit and ACR. **RHR:** [a] R^2 = 0.352, adjusted R^2 = 0.335. [b] R^2 = 0.383, adjusted R^2 = 0.357. **RR interval:** [c] R^2 = 0.290, adjusted R^2 = 0.271. [d] R^2 = 0.327, adjusted R^2 = 0.298.

Supplementary Table S10: Independent associations with atrial electrical activity.

	Independent variable	Standardised β (95%CI)	*p*-value
**P wave duration**			
Total study population[a]	Hb	0.267 (0.036 to 0.498)	0.024
HIV+/ART+[b]	ALT	0.538 (0.226 to 0.851)	0.001
	RHR	-0.323 (-0.516 to -0.085)	0.009
	Viral load	0.306 (0.018 to 0.594)	0.038
**PR interval**			
Total study population[c]	BMI	0.381 (0.157 to 0.605)	0.001
	Sex (women)	-0.240 (-0.468 to -0.012)	0.040
HIV+/ART+[d]	BMI	0.407 (0.103 to 0.710)	0.010
**PR segment**			
Total study population[e]	RHR	-0.235 (-0.451 to -0.019)	0.033
HIV+/ART+[f]	Triglycerides	0.373 (0.049 to 0.697)	0.025
	RHR	-0.252 (-0.501 to -0.003)	0.047

Model B (variables considered): variables included in model A, SBP, DBP, TC, HDL, LDL, TG, fasting glucose, HbA1c, haematocrit and ACR. P wave duration: [a] R^2 = 0.066, adjusted R^2 = 0.054. [b] R^2 = 0.284, adjusted R^2 = 0.238. PR interval: [c] R^2 = 0.151, adjusted R^2 = 0.128. [d] R^2 = 0.131, adjusted R^2 = 0.113. PR segment: [e] R^2 = 0.059, adjusted R^2 = 0.046. [f] R^2 = 0.165, adjusted R^2 = 0.129.

Supplementary Table S11: Independent associations with ventricular electrical activity.

	**Independent variable**	**Standardised β (95%CI)**	***p*-value**
	**QRS complex**		
Total study population[a]	SBP	0.301 (0.088 to 0.514)	0.006
HIV+/ART+[b]	SBP	0.367 (0.061 to 0.673)	0.020
	**ST segment**		
Total study population[c]	RHR	-0.405 (-0.595 to -0.214)	< 0.001
	Sex (women)	0.508 (0.308 to 0.708)	< 0.001
	Age	0.234 (0.038 to 0.430)	0.020
	LDL	-0.224 (-0.415 to -0.033)	0.022
HIV+/ART+[d]	Sex (women)	0456 (0.208 to 0.704)	0.001
	RHR	-0.367 (-0.603 to -0.131)	0.003
	ACR	0.222 (0.006 to 0.439)	0.044
	**ST interval**		
Total study population[e]	RHR	-0.474 (-0.633 to -0.286)	< 0.001
	Haematocrit	0.395 (0.193 to 0.598)	< 0.001
	Sex (women)	0.328 (0.120 to 0.537)	0.002
	LDL	-0.209 (-0.404 to -0.015)	0.035
HIV+/ART+[f]	RHR	-0.442 (-0.684 to -0.201)	0.001
	Hb	0.441 (0.197 to 0.684)	0.001
	HIV duration (> 5 years)	0.270 (0.003 to 0.537)	0.047
	**T wave duration**		
Total study population[g]	SBP	0.419 (0.228 to 0.609)	< 0.001
	Sex (women)	-0.219 (-0.413 to -0.025)	0.027
	RHR	-0.229 (-0.417 to -0.041)	0.018
	ALT	0.230 (0.023 to 0.438)	0.030
HIV+/ART+[h]	SBP	-0.487 (0.214 to 0.761)	0.001
	Hb	0.303 (0.074 to 0.531)	0.011
	Fasting glucose	0.244 (0.010 to 0.477)	0.041
	**QT interval**		
Total study population[i]	RHR	-0.580 (-0.765 to -0.400)	< 0.001
	Haematocrit	0.276 (0.026 to -0.388)	0.005
	Sex (women)c	0.158 (0.004 to 0.363)	0.008
HIV+/ART+[j]	RHR	-0.547 (-0.775 to -0.319)	< 0.001
	Hb	0.258 (0.030 to 0.486)	0.027
	Alcohol consumption (yes)	0.256 (0.021 to 0.490)	0.033

Model B (variables considered): variables included in model A, SBP, DBP, TC, HDL, LDL, TG, fasting glucose, HbA1c, haematocrit and ACR. QRS complex: [a] $R^2 = 0.096$, adjusted $R^2 = 0.084$. [b] $R^2 = 0.108$, adjusted $R^2 = 0.089$. ST segment: [c] $R^2 = 0.406$, adjusted $R^2 = 0.373$. [d] $R^2 = 0.387$, adjusted $R^2 = 0.347$. ST interval: [e] $R^2 = 0.410$, adjusted $R^2 = 0.377$. [f] $R^2 = 0.435$, adjusted $R^2 = 0.398$. T wave duration: [g] $R^2 = 0.367$, adjusted $R^2 = 0.322$. [h] $R^2 = 0.461$, adjusted $R^2 = 0.414$. QT interval: [i] $R^2 = 0.436$, adjusted $R^2 = 0.413$. [j] $R^2 = 0.474$, adjusted $R^2 = 0.439$.

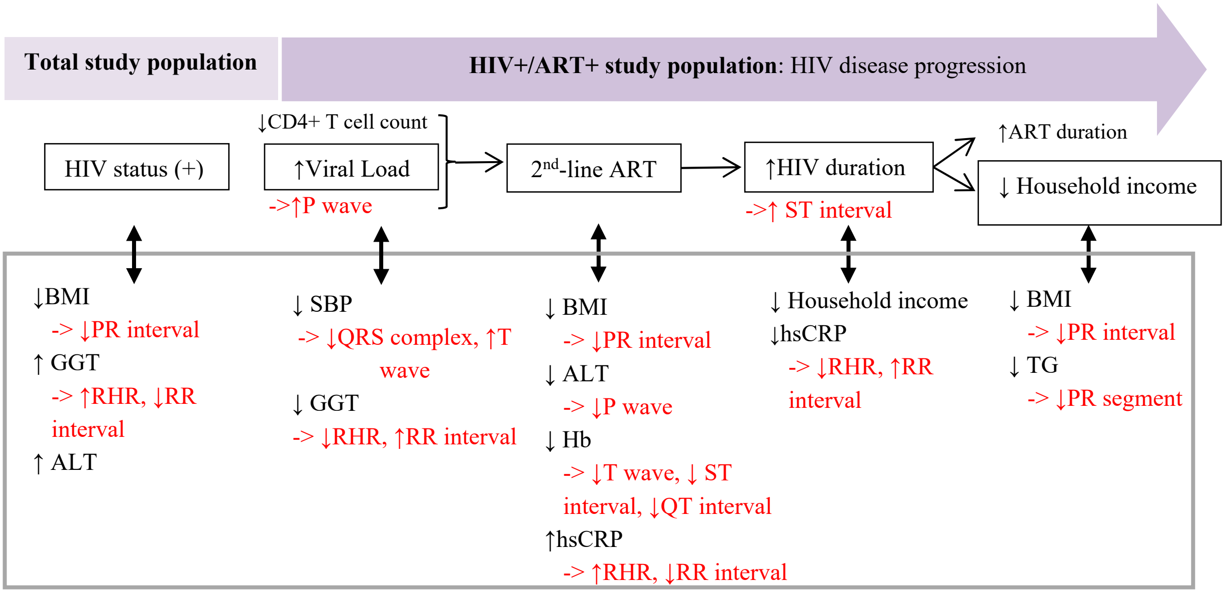

Supplementary Figure S1: Summary of regression results - independent associations of cardiometabolic and ECG variable outcomes with HIV and ART. HIV – human immunodeficiency virus, ART – antiretroviral therapy, BMI – body mass index, GGT – gamma-glutamyl transferase, RHR – resting heart rate, ALT – alanine aminotransferase, SBP – systolic blood pressure, Hb – haemoglobin, hsCRP – high sensitivity C-reactive protein, TG – triglycerides, ↓ – decreased/lower, ↑ – increased/higher. Black points (in the box): cardiometabolic variables and socioeconomic status independently associated with markers of disease progression. Red points: ECG variable outcomes associated with cardiometabolic variables and markers of disease progression.

Supplementary Figure S1: Summary of regression results
